# Absorption, Thermal Relaxation Time, and Beam Penetration Depth of Laser Wavelengths in Ex Vivo Porcine Gingival Tissues

**DOI:** 10.3390/dj13090397

**Published:** 2025-08-29

**Authors:** Mayssaa Ismail, Thibault Michel, Daniel Heysselaer, Saad Houeis, Andre Peremans, Alain Vanheusden, Samir Nammour

**Affiliations:** 1 Department of Dental Science, Faculty of Medicine, University of Liege, 4000 Liege, Belgium; dr.mayssaaism@outlook.com (M.I.); thibaultmichel@hotmail.fr (T.M.); daniel.heysselaer@gmail.com (D.H.); saad.houeis@gmail.com (S.H.); alain.vanheusden@ulg.ac.be (A.V.); 2 Department of Physics and Photonics, Laserspec Inc., 5020 Namur, Belgium; andre.peremans@laserspec.be

**Keywords:** laser, tissue interaction, absorption, thermal relaxation time, light transmittance, light attenuation

## Abstract

**Background/Objectives**: The laser beam absorption and thermal relaxation time (TRT) in oral tissues are key to optimizing treatment parameters. The aim of this study is to (1) evaluate, in an ex vivo study, the percentage of attenuation and transmittance of each wavelength as a function of tissue thickness; (2) determine the global absorption coefficient, α, of pig gingival tissue for the most commonly used wavelengths in dentistry; (3) calculate the thermal relaxation time (TRT) of oral tissue for these wavelengths; and (4) determine their corresponding penetration depths. **Methods:** We measured the transmission of different laser wavelengths through pig oral gingival tissues (Mandibular labial gingiva). We placed each tissue sample between two glass slides with minimal light attenuation. The input and output powers were measured after irradiating the tissue at different specific wavelengths: 450 nm, 480 nm, 532 nm, 632 nm, 810 nm, 940 and 980 nm, 1064 nm, 1341, 2780 nm and 2940 nm. After calculating the transmittance values, we plotted transmittance curves for each wavelength. Using the Beer–Lambert law, we then calculated the absorption coefficient (α) of each wavelength in the oral gingival tissue. Absorption coefficients were then used to calculate the TRT and penetration depth for each wavelength. **Results:** Among the tested wavelengths, 810 nm exhibited the lowest absorption in ex vivo porcine gingival tissue (α = 9.60 cm^−1^). The 450 nm blue laser showed moderate absorption (α = 26.8 cm^−1^), while the Er:YAG laser at 2940 nm demonstrated the highest absorption (α = 144.8 cm^−1^). We ranked the wavelengths from most absorbed to least absorbed by porcine oral gingival mucosa as follows: 2940 nm > 2780 nm > 450 nm > 480 nm > 532 nm > 1341 nm > 632 nm > 940 nm > 980 nm > 1064 nm > 810 nm. **Conclusions:** Absorption and the TRT vary significantly across wavelengths. Erbium lasers are characterized by the highest absorption and minimal light penetration. Infrared diodes, particularly the 810 nm wavelength, showed the lowest absorption and deepest tissue penetration and exhibited the highest thermal relaxation time. The 480 nm laser demonstrated greater absorption by porcine gingival tissue compared to the 532 nm laser. These findings provide evidence-based guidance for wavelength selection in dental treatments and photobiomodulation, enabling improved precision, safety, and therapeutic efficacy in clinical practice.

## 1. Introduction

Laser systems emitting coherent radiation at defined wavelengths are widely used in dentistry for both diagnostic and therapeutic procedures [1]. Despite their widespread adoption, the determination of the optimal laser wavelength for specific clinical applications remains a subject of ongoing debate. This uncertainty is perpetuated by inconsistencies across basic research and clinical studies, which underscore the complex interactions between laser energy and biological tissues [2].

When laser light is applied to the surface of the skin, approximately 4–7% of the incident energy is reflected at the interface due to the difference in refractive indices between air and tissue [3]. The transmission of the remaining light through the skin and underlying subcutaneous tissues is influenced by both absorption and scattering phenomena. The magnitude of these effects is determined by the histological characteristics of the tissue and the physical properties of the laser light, particularly its wavelength [4,5].

It is crucial to emphasize that effective photobiomodulation (PBM) therapy targeting deeper oral tissues, such as bone, necessitates the use of wavelengths with sufficient penetration depth to ensure adequate energy delivery to the intended site. Given that the therapeutic efficacy of PBM is highly dose-dependent, the selection of an appropriate wavelength capable of reaching the target tissue is essential. Several studies have demonstrated that the optical properties of biological tissues (absorption and scattering) vary significantly with wavelength, thereby influencing the spatial distribution of light energy within the tissue. Consequently, a comprehensive understanding of the wavelength-dependent penetration and absorption characteristics in oral tissues is fundamental to the optimization of PBM treatment protocols [6].

A thorough understanding of the physical properties of laser light and its interactions with biological tissues is essential for enhancing the efficacy and quality of clinical treatments across a range of applications [7]. In the context of targeting deep oral tissues, it is particularly and clinically relevant to account for the attenuation of light intensity as it propagates through oral gingival tissues. This attenuation process is governed by the principles of the Beer–Lambert law, which describes the exponential decrease in light intensity as a function of tissue depth and optical properties of the light. Achieving a therapeutic effect therefore requires the precise delivery of an appropriate wavelength with sufficient energy to reach the target tissue, underscoring the critical importance of light penetration in treatment planning.

Although the existing literature provides extensive separate data on the specific absorption characteristics of laser light in water [8], hemoglobin [9], collagen [10], protein [11], skin [12], and pigmented tissues [13], few have focused specifically on the absorption characteristics of different laser wavelengths in oral gingival mucosa. This lack of data limits our ability to optimize laser parameters for clinical dental applications. This study addresses that gap by providing quantitative absorption, penetration depth, and thermal relaxation time data across commonly used wavelengths in dentistry.

A robust understanding of laser–tissue interactions within the oral cavity is critical for optimizing clinical outcomes and doses while minimizing the risks of thermal injury and subtherapeutic effects [14].

This ex vivo study aims to evaluate the absorption coefficient, α, penetration depth, δ, and thermal relaxation time (TRT) of porcine gingival tissue when exposed to clinically relevant dental laser wavelengths.

The null hypothesis (H_0_) posits no statistically significant differences in absorption among the tested wavelengths.

## 2. Materials and Methods

### 2.1. Sample Collection and Preparation

No animals were sacrificed for the purpose of this study. All biological samples were collected from the heads of animals that had been freshly slaughtered at a licensed abattoir for routine commercial meat production. Samples were collected immediately to preserve tissue integrity, prevent dehydration, and avoid any structural deformation that could bias the results. Since the research is an ex vivo study and did not involve patients or live animals, prior approval from the University Ethics Committee was not required.

**Figure 1:**
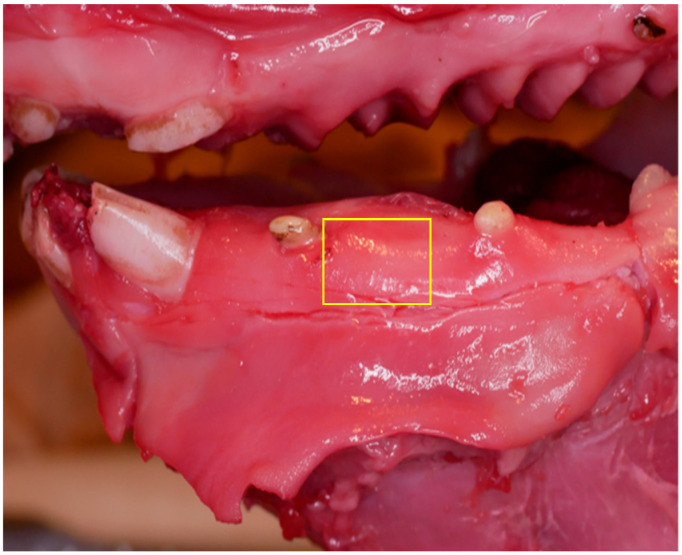
Vestibular gingival region of a porcine head showing the area of the excised samples.

Fresh porcine heads were obtained, and a total of 30 soft tissue samples were harvested from the vestibular gingival region of the lower jaw, specifically at the level of the molars and premolars. Samples were collected 1 mm apical to the cervical margin, each measuring approximately 1.5 cm × 1.5 cm in surface area (Figure 1). These full-thickness specimens primarily consisted of attached gingiva, though the inclusion of free gingiva was also possible. Samples with excessive pigmentation or visible damage were excluded. Only fresh, non-pigmented, healthy tissue was used to minimize color-related absorption bias. The samples were distributed across six different thickness categories: 0.50 mm, 0.57 mm, 0.73 mm, 0.75 mm, 0.85 mm, and 1.00 mm.

Each sample was irradiated with only one specific laser wavelength to prevent cumulative thermal effects or cross-exposure bias. For each of the 11 tested wavelengths, five independent samples were used (*n* = 5), ensuring that each sample was used for a single measurement only.

All experiments were conducted under controlled room conditions (22 ± 1 °C, 50% relative humidity) to ensure consistency in optical measurements.

**Figure 2:**
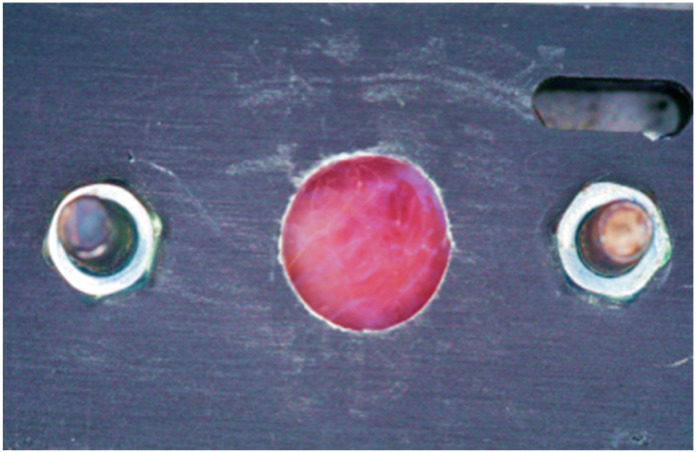
Tissue sample positioned between two FOCtek optical glass slides within a custom metal stabilization device.

All tissues were stored in saline-moistened sterile gauze and processed within 2 h post excision to minimize dehydration and maintain consistent optical properties. We mounted the samples between two Foctek optical glass slides (FOCTEK IN140728-15, Foctek Inc., Fujian, China) within a custom metal stabilization device (Figure 2).

The glass ensures minimal light absorption (~10%) during transmission measurements. As our research did not involve any human or animal study, it does not require a prior approval from the ethical committee of the University of Liège.

### 2.2. Tissue Thickness Measurement and Classification

**Figure 3:**
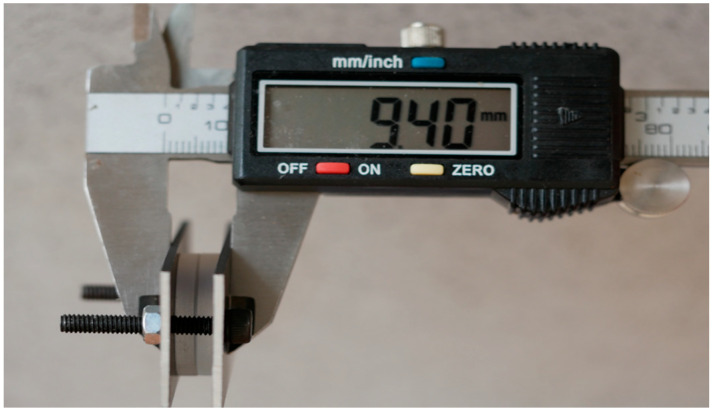
Measurement of tissue sample thickness using calibrated electronic micrometer.

We determined the full thickness of each tissue sample using a calibrated electronic micrometer with ±10 μm accuracy (H0075, XiamenSizhi, Huli, China) (Figure 3) with an accuracy of ±10 μm. The actual thickness of the tissue sample was determined by subtracting the combined thickness of the glass slides and the metal stabilization frame from the total measured thickness.

A total of thirty samples with varying thicknesses were collected for analysis: 0.50 mm, 0.57 mm, 0.73 mm, 0.75 mm, 0.85 mm, and 1.00 mm.

### 2.3. Laser Source and Wavelength Generation

Laser irradiation was conducted across a broad spectrum of wavelengths commonly utilized in dental applications. For wavelengths ranging from 410 to 2640 nm, a visible-range Optical Parametric Oscillator (OPO) system (VIS-OPO Laserspec SRL, Malonne, Namur, Belgium) constructed around a β-Barium Borate (BBO) crystal, and calibrated monthly (±2% tolerance) was employed. Each sample (*n* = 5 per wavelength) was irradiated with a power of 100 mW, spot size 1 mm, under controlled temperature (22 °C) and humidity (50%). This configuration enabled the generation of discrete wavelengths via phase matching adjustments of the nonlinear crystal.

Specifically, wavelengths 450, 480, 532, 632, 810, 940, 980, 1064, and 1341 nm were produced using the visible OPO system.

For longer wavelengths (2780 nm and 2940 nm), we used an infrared OPO system (MIR-OPO Laserspec SRL, Malonne, Namur, Belgium) built around a lithium niobate (LiNbO_3_) crystal.

The OPO system allows the precise control of wavelength via nonlinear crystal modulation, enabling us to select laser output from 200 to 600 nm (visible) and via filters for infrared extensions (2780–2940 nm).

### 2.4. Laser Irradiation Parameters

For each tested wavelength, tissue samples were irradiated using standardized parameters to ensure consistency across measurements. The laser output power was set at 100 mW, with a spot size of 1 mm diameter (area = 0.00785 cm^2^), resulting in a power density of approximately 12.74 W/cm^2^.

All irradiations were performed in continuous wave (CW) mode, with each exposure lasting 5 s. This corresponds to a total energy delivery of 0.5 J per sample and an energy density (fluence) of approximately 63.7 J/cm^2^.

The laser beam was directed perpendicularly to the tissue sample surface, with the fiber tip in contact with the upper glass slide, ensuring minimal beam divergence and uniform irradiation.

### 2.5. Irradiation Procedure and Energy Measurement

We measured the effective energy delivered into soft tissues using a calibrated Gentec-EO power meter (Gentec-EO, Québec City, QC, Canada) (±2% tolerance), which was calibrated monthly according to the manufacturer’s standards.

The optical glass slides (FOCTEK IN140728-15, Foctek Inc., Fujian, China) used in the experimental setup exhibit wavelength-dependent transmittance values. According to the manufacturer specifications, these slides transmit approximately 93.0% of incident light in the visible to near-infrared range (400–1500 nm) and around 80.0% in the mid-infrared range (2700–3000 nm).

To ensure the accurate calculation of transmittance and absorption coefficients, the measured input power (Pin_measured) was corrected for glass transmission losses. The corrected input power (Pin_corrected) was calculated using the following formula:

Pin_corrected = Pin_measured/T_glass

**Figure 4:**
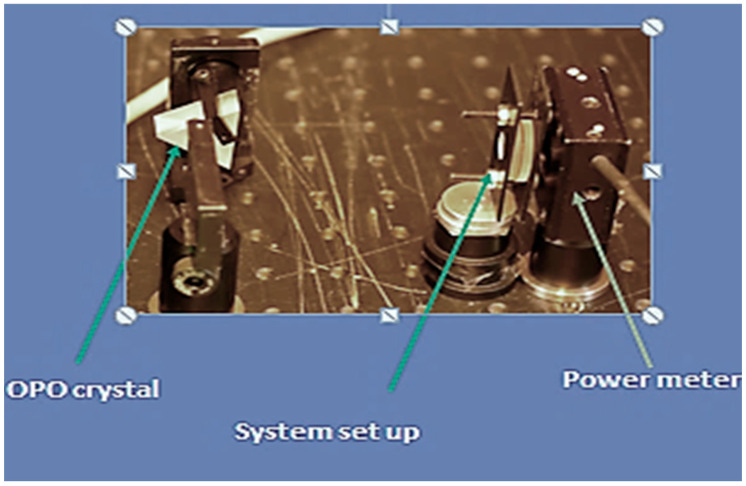
A view of the experimental setup measuring the transmitted energy through tissue samples.

Following sample placement, the calibrated incident beam (P_in_) and the output power (P_out_) were measured (Figure 4) and the percentage of the beam absorbed by tissue was then calculated using the following formula:

T = (P_out_/P_in_) × 100

The absorbance (A) of each sample was determined as

A = 100 − T

### 2.6. Baseline Reference Measurements (Control Setup)

To ensure the accurate calculation of transmittance and absorption coefficients, baseline control measurements were performed by measuring laser transmission through the optical setup without tissue (glass slides only). This allowed the precise correction of input power (Pin) for inherent attenuation by the glass. These reference values were recorded for each wavelength and used to normalize the transmission measurements across all tissue samples.

Each wavelength was tested on five independent tissue samples of varying thickness to ensure internal consistency.

### 2.7. Calculation of Absorption Coefficients, Thermal Relaxation Time and the Penetration Depth

After calculating the transmittance values, we plotted transmittance curves for each wavelength. Using the Beer–Lambert law, we calculated the absorption coefficient (α) for each wavelength according to the following equation:

T = exp(−α × d)

where T is the transmittance, α is the absorption coefficient, and d is the tissue thickness. By rearranging the equation, the absorption coefficient was determined as

α = −ln(T)/d

To minimize errors, each measurement was repeated three times per wavelength per sample, and the average was calculated.

Once the absorption coefficients (α) were obtained, we calculated the penetration depth (δ,) in the pig’s oral gingival tissue for each wavelength as the inverse of the absorption coefficient (e.g., a wavelength of 450 nm with an absorption coefficient of α = 26.75 cm^−1^ yielded a penetration depth of approximately = 1/26.75 = 0.037 cm = 370 µm).

δ, = 1/α

Finally, we obtained the thermal relaxation time (TRT) for the oral gingival tissue using the following formula:

TRT = δ^2^/(4 × K)

where K is the thermal diffusivity of the tissue. The TRT represents the time required for the tissue to dissipate 63% of heat following laser irradiation. The TRT is critical in assessing the tissue’s ability to dissipate heat between laser pulses, thereby influencing the risk of tissue overheating and thermal damage.

### 2.8. Analyzed Wavelengths

Measurements were conducted using an automated acquisition system at 2 nm intervals across the ranges of 420–1532 nm and 2700–3000 nm. The primary wavelengths analyzed in this study included the following:

- Visible range: 450 nm (blue), 480 nm (Argon), 532 nm (KTP), and 632 nm (HeNe).
- Near-infrared range: 810 nm, 940 nm, 980 nm (diodes), 1064 nm (Nd:YAG), and 1341 nm (Nd:YAP).
- Mid-infrared range: 2780 nm (Er,Cr:YSGG) and 2940 nm (Er:YAG).

These wavelengths were selected due to their clinical relevance in various dental disciplines, including restorative dentistry, periodontology, endodontics, surgery, and implantology.

## 3. Statistical Analysis

Statistical analyses were achieved using Prism 5^®^ software (GraphPad Software, Inc., San Diego, CA, USA). *p* < 0.05 was considered statistically significant. The confidence level of the study was proposed to be 95% with *p* < 0.01. Descriptive statistics, including the means and standard deviations, were calculated. Paired ANOVA tests coupled with the Newman–Keuls multiple comparison test (post hoc test) were used.

## 4. Results

**Figure 5:**
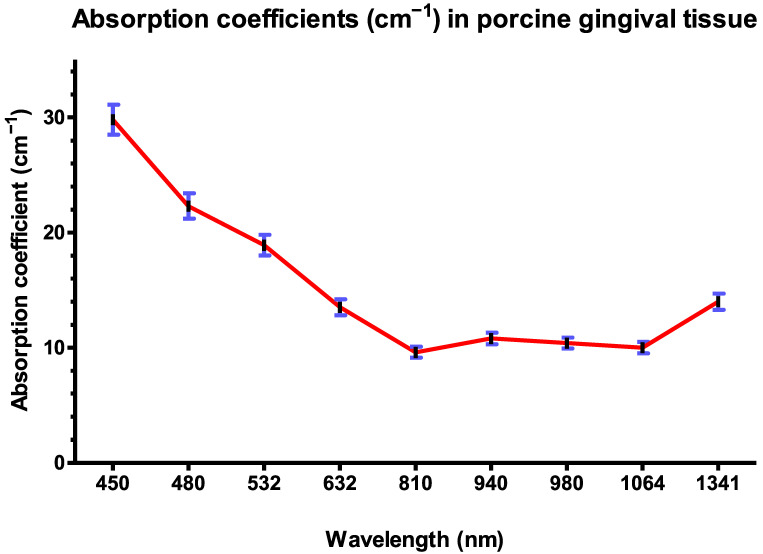
Absorption coefficients (cm^−1^) of wavelengths 450–1341 nm in porcine gingival tissue.

Table 1 and Figure 5 show that at shorter wavelengths in the visible spectrum, such as at 450 nm and 480 nm, the absorption coefficients are relatively high, with respective values of 26.8 cm^−1^ and 22.3 cm^−1^, indicating a good absorption of light by the porcine gingival tissues. As the wavelength increases within the visible range from 532 nm to 632 nm, there is a trend of decreasing absorption coefficients (α) from 19 cm^−1^ to 13.5 cm^−1^, suggesting the reduced absorption of light in this range.

In the near-infrared range, wavelengths of 810 nm to 1064 nm exhibit relatively the lowest α, with an average of 10.2 cm^−1^. However, there is a slight increase in α at 940 nm compared to 810 nm, indicating a localized peak in absorption efficiency around this wavelength with an α coefficient of absorption of 10.8 cm^−1^. These coefficients of absorption tend to increase upon increasing the wavelength to 14 cm^−1^ for a laser wavelength of 1341 nm.

At longer wavelengths beyond the near-infrared range, particularly at 2780 nm and 2940 nm, there are significant peaks in the absorption coefficient with simultaneous values of 142.3 cm^−1^ and 144.8 cm^−1^ (Table 1).

After calculating the absorption coefficients of the different wavelengths, we can rank the wavelengths from most absorbed to least absorbed by porcine gingival tissues as follows:

2940 nm > 2780 nm > 450 nm > 480 nm > 532 nm > 1341 nm > 632 nm > 940 nm > 980 nm > 1064 nm > 810 nm.

The absorption coefficients from highest to lowest reveal that the porcine gingival tissues exhibit the highest absorption at wavelengths of 2940 nm and 2780 nm, with α values of 144.8 cm^−1^ and 142.3 cm^−1^, respectively. Following these values, the absorption decreases gradually with decreasing wavelengths, showcasing significant absorption at 450 nm (26.8 cm^−1^) and 480 nm (22.3 cm^−1^) in the visible spectrum. Further down the spectrum, absorption diminishes, with absorption coefficients decreasing to 19 cm^−1^ at 532 nm and 13.5 cm^−1^ at 632 nm. In the near-infrared range, wavelengths of 1341 nm exhibit an α of 14 cm^−1^, while a localized peak is observed at 940 nm with an α of 10.8 cm^−1^. We observed the lowest absorption at the wavelength of 810 nm, with the lowest α of 9.6 cm^−1^. (Table 1 and Figure 5).

ANOVA statistical analysis revealed significant differences in absorption coefficients among the tested wavelengths (*p* < 0.01). On the other hand, we observed that both 2940 nm and 2780 nm wavelengths had a significantly higher significant difference than all other wavelengths (*p* < 0.001).

**Figure 6:**
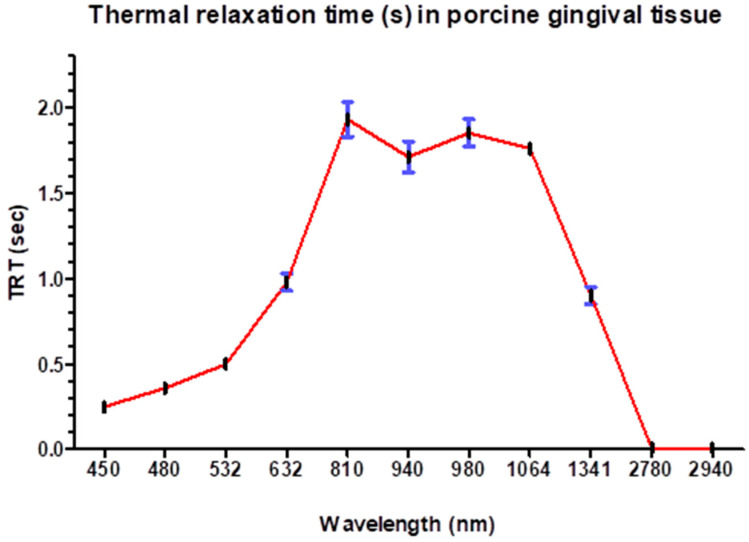
Thermal relaxation time (s) of different wavelengths in porcine oral soft tissues.

Table 2 and Figure 6 represent a set of measurements of the thermal relaxation time (TRT) in seconds for various wavelengths in nanometers (nm).

At shorter wavelengths in the visible spectrum, such as 450 nm and 480 nm, the thermal relaxation times are relatively low, with values of 0.249 s and 0.357 s, respectively. As the wavelength increases within the visible range from 532 nm to 632 nm, there is a trend of increasing thermal relaxation time from 0.495 s to 0.984 s.

In the near-infrared range, as the wavelength increases from 810 nm to 1064 nm, there is a decrease in the thermal relaxation time ranging from 1.93263 s to 1.755 s. Notably, there is a slight decrease in the TRT at 1341 nm compared to 1064 nm, with a value of 0.904 s.

At longer wavelengths beyond the near-infrared range, particularly at 2780 nm and 2940 nm, the TRT significantly decreases to 0.009 s and 0.008 s, respectively, suggesting rapid heat diffusion at these wavelengths.

So, the tissue with thickness between 0.5 mm and 3 mm dissipates heat approximately 227.74 times faster when irradiated by the laser at 2940 nm compared to when irradiated by the laser at 810 nm.

**Figure 7:**
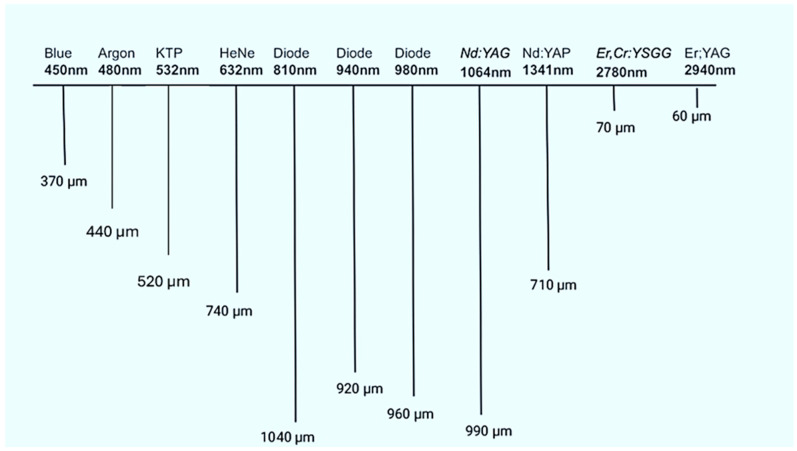
Schema of beam penetration depth (µm) of different wavelengths in porcine oral gingival tissues.

Table 3 and Figure 7 show that at shorter wavelengths in the visible spectrum, such as 450 nm and 480 nm, the depth of beam penetration is relatively low, with values of 0.37 mm and 0.66 mm, respectively. As the wavelength increases within the visible range from 532 nm to 632 nm, there is a trend of a slight increase in the depth of penetration, with values ranging from 0.52 mm to 0.74 mm.

In the near-infrared range, wavelengths from 810 nm to 1064 nm exhibit further increases in penetration depth, with values ranging from 0.99 mm to 1.04 mm. This indicates that near-infrared wavelengths penetrate deeper into porcine soft tissues compared to visible light wavelengths.

At longer wavelengths beyond the near-infrared range, particularly at 2780 nm and 2940 nm, the depth of penetration significantly decreases to 0.07 mm and 0.06 mm, respectively. This suggests that these longer wavelengths are strongly absorbed by porcine soft tissues, resulting in very limited penetration.

Overall, the data reveal a wavelength-dependent trend in both the depth of penetration and the attenuation of 99% of the light. Shorter wavelengths between 450 nm and 1341 nm are less absorbed and therefore penetrate more deeply, with an average penetration depth of 0.76 mm. In contrast, longer wavelengths such as 2780 nm and 2940 nm are highly absorbed and remain more superficial, with an average penetration depth of 0.065 mm.

Our results demonstrate a statistically significant difference in the absorption characteristics of porcine gingival tissue across the tested laser wavelengths. Therefore, the null hypothesis (H_0_), which posits no significant difference in absorption among the wavelengths, is rejected.

## 5. Discussion

### 5.1. Clinical Relevance of Laser–Tissue Interaction

A comprehensive understanding of the nature of light, tissue interaction could simplify and ease the choice of treatment parameters.

In this study, we utilized porcine gingival mucosa because gingival mucosa is the most irradiated area in the human mouth. Cilesiz and Welch highlighted that inevitable tissue deformation and handling, including processes like drying, freezing, and dehydration, can significantly impact the optical properties of the tissue [15]. To mitigate these effects, we exclusively utilized fresh red soft tissue that was transported in a gauze soaked with physiological fluid to prevent water loss. Furthermore, we conducted the measurements with minimal handling to ensure the integrity of the samples within 2 h to preserve color, dehydration, and tissue integrity.

Based on their high absorption and minimal penetration depth, Erbium lasers (2940 nm and 2780 nm) are ideal for precise soft tissue ablation, such as cavity preparation or delicate esthetic surgical procedures. Blue lasers (450–480 nm), due to their stronger absorption and shorter thermal relaxation times compared to 810–980 nm wavelengths, are more suited for soft tissue surgical applications in the oral cavity.

Diode lasers (810–980 nm), with their deeper penetration, are well suited for applications involving deeper oral tissues, such as photobiomodulation (PBM) of deep tissues, and for blood coagulation and stabilization in post-extraction bone sites.

### 5.2. Experimental Design and Model Considerations

To irradiate the tissues, we utilized an optical parametric oscillator (OPO) laser, which generates wavelengths between 210 nm and 4500 nm. This OPO laser was chosen for its ability to deliver a wide range of wavelengths, effectively producing coherent light at wavelengths significantly different from the pump laser [16].

For accuracy, the light intensity attenuation caused by the glass supporting the soft tissues during irradiation was measured and subtracted from our calculations before determining tissue-specific absorption values.

The selection of porcine oral tissue in this study was based on its well-documented anatomical and physiological similarities to human oral tissues. Previous studies have demonstrated that porcine gingiva closely resembles human gingiva in both structure and composition, making it a suitable model for investigating laser–tissue interactions [17].

### 5.3. Light Attenuation, Absorption, and Penetration Concepts

In the context of this study, attenuation refers to the reduction in light energy as it passes through tissue. It includes the portion of light that is absorbed, reflected, or refracted, thus restricting its further propagation and penetration within the tissue.

Our approach assumes that attenuation approximates total absorption. However, we did not directly measure light scattering, which may also contribute to overall attenuation.

The precise quantification of beam scattering, refraction, and the amount of light absorbed by the tissue were not addressed in this study and remain topics for future research. Nevertheless, the relative levels of light attenuation observed across the various tested tissues may serve as indicators of the overall relative amounts of light absorbed and attenuated by these tissues [18].

### 5.4. Key Findings and Interpretation (Absorption, TRT, Penetration)

The purpose of our study was to assess the depth of light penetration and the extent of attenuation for various wavelengths, 450 nm (blue), 480 nm (Argon), 532 nm (KTP), 632 nm (HeNe), 810 nm (diode), 940 nm (diode), 980 nm (diode), 1064 nm (Nd:YAG), 1341 nm (Nd:YAP), 2780 nm (Er,Cr:YSGG), and 2940 nm (Er:YAG), through porcine oral gingiva samples of varying thicknesses ranging from 0.5 mm to 3 mm.

Our results show that Erbium lasers (Er,Cr:YSGG and Er:YAG) have the highest absorption coefficients (α) among the tested wavelengths, explaining their rapid and efficient ablation capabilities. This high absorption also accounts for the limited penetration depth of Erbium lasers. Consequently, most photons emitted by these lasers are absorbed at the tissue surface, resulting in significant tissue ablation, while only a few photons penetrate into the subsurface layers, explaining the minimal coagulation effect associated with Erbium lasers.

### 5.5. Thermal Relaxation Time and Heat Management

Considering thermal relaxation time (TRT), our results showed that Erbium lasers have the shortest TRT, indicating rapid heat dissipation from the irradiated tissue. Consequently, these wavelengths cause less thermal damage to adjacent tissues compared to other lasers.

For photobiomodulation (PBM), the 810 nm diode laser is preferred for treating deeper tissues due to its minimal absorption and greater penetration depth in oral tissues.

Interestingly, lasers with intermediate absorption coefficients, such as blue lasers at 450 nm, demonstrate higher absorption than diode lasers at 810 and 980 nm. By extrapolation, the 450 nm laser may achieve more efficient cutting and outperform Erbium lasers in coagulation because of its longer TRT. This versatility should be considered in daily dental practice to tailor the choice of wavelength to each surgical procedure.

Formerly known as Low-Level Laser Therapy (LLLT), photobiomodulation (PBM) therapy involves applying low-power laser or LED light to promote tissue repair, reduce inflammation, and induce analgesia [19,20]. Both red and infrared lasers are used in PBM, but they can have different effects on soft tissues and bone due to variations in penetration depth and biological response [21].

Diode red lasers or HeNe lasers at 632 nm are commonly used in PBM for their superficial tissue penetration, making them effective for treating wounds, inflammation, and superficial pain in soft tissues [19,20].

In contrast, infrared (IR) lasers have longer wavelengths, typically ranging from approximately 700 nm to 1200 nm [22]. According to our results, these wavelengths penetrate deeper into tissues, reaching structures such as muscles, tendons, salivary glands, and even bone. Therefore, IR lasers, such as diode lasers at 810 nm, 940 nm, and 980 nm, are often used in PBM to promote bone healing and regeneration, support salivary gland function, and manage deeper musculoskeletal conditions [23].

The 810 nm wavelength, with the greatest tissue penetration depth of approximately 1.04 mm, is well suited for targeting subepithelial structures such as connective tissue, blood vessels, and nerve endings, making it effective for photobiomodulation in deep tissue healing and bone regeneration. In contrast, the 2940 nm wavelength, with the lowest penetration depth of just 0.06 mm, is ideal for precise epithelial or skin ablation with minimal thermal diffusion, making it suitable for procedures such as skin or gingival reshaping and cavity preparation. Additionally, blue lasers (e.g., 450 nm) may offer enhanced cutting effects compared to infrared lasers (810–980 nm), making them advantageous for soft tissue incisions and gingival depigmentation.

Several lasers used in oral surgery exhibit a high thermal relaxation time (TRT). Our results showed that the diode laser at 810 nm has a TRT of 1.93 s, meaning that the irradiated tissue requires a relatively long time to dissipate the accumulated heat. Consequently, using this laser during prolonged irradiation carries a greater risk of cumulative heat buildup when used in continuous or high-frequency pulsing. Without sufficient intervals between pulses, this may lead to unintended collateral thermal damage. To avoid tissue damage and reduce surgery time, the interval between pulses should exceed the TRT to allow sufficient heat dissipation. Therefore, it is suggested to use very short pulses in the nanosecond range, separated by resting times in microseconds, to minimize heat buildup and ensure safer irradiation during delicate surgical procedures. This approach follows the thermal confinement principle described by Niemz, which states that energy must dissipate before the next pulse to prevent tissue heat damage [4].

The literature emphasizes the importance of activating (initiating) the fiber tip prior to using diode lasers (810–980 nm) in oral surgery. This activation procedure typically involves touching the edge of the fiber tip, before irradiation, to a dark, absorbent material such as black articulating paper, cork, or another dark substance. This contact produces a “charring” effect on the tip. The resulting carbonization around the activated tip selectively absorbs the laser light and generates a high local temperature at the fiber’s edge, enabling the effective vaporization of the underlying tissue [24]. The activation of the laser fiber thus enhances the cutting efficiency of the specific wavelength used [25].

### 5.6. Clinical Decision-Making Based on Optical Properties

The absorption coefficients, thermal relaxation times (TRTs), and beam penetration depths measured in this study provide a foundation for selecting appropriate laser wavelengths for specific clinical applications.

- Erbium lasers (2940 nm and 2780 nm) demonstrate high absorption and an extremely short TRT, making them ideal for precise surface ablation procedures, such as epithelial or skin peeling, gingival contouring, where minimal thermal spread is essential.
- Diode lasers (810 nm and 980 nm), characterized by low absorption and a high TRT, are well suited for deep tissue photobiomodulation (PBM) and coagulation. Their deeper penetration allows energy delivery to subepithelial and even osseous structures.
- Blue diode lasers (450–480 nm) exhibit moderate oral tissue absorption, making them effective for coagulation, incision, and soft tissue depigmentation.
- Intermediate wavelengths such as 532 nm, 632 nm, and 1341 nm offer a balance between absorption and penetration depth, allowing the moderate coagulation and treatment of oral soft tissues without significant heat propagation.

To further guide clinical application, penetration depth thresholds can serve as a decision-making framework:

- Shallow penetration (0.06–0.08 mm): Optimal for highly esthetic and superficial treatments (e.g., Erbium lasers in superficial epithelial ablation).
- Moderate penetration (0.3–0.6 mm): Suitable for mucosal treatments (e.g., 450–532 nm).
- Deep penetration (≥1 mm): Required for PBM and deep submucosal interventions (e.g., 810–980 nm).

Thermal safety must also be considered. For lasers with a long TRT (e.g., 810 nm: TRT = 1.93 s), clinical use should employ short pulse durations and low duty cycles to allow adequate tissue cooling and prevent cumulative heat damage. Pulsed emission modes are recommended over continuous wave (CW) settings in such cases.

### 5.7. Clinical Relevance

The absorption properties, thermal relaxation times (TRTs), and beam penetration depths of oral tissues represent fundamental factors that must be carefully considered when selecting the most suitable laser wavelength and determining the ideal irradiation parameters for each specific clinical procedure. These tissue-specific characteristics directly influence how laser energy interacts with the target area, affecting both the efficacy and safety of the treatment. A comprehensive understanding of these variables enables clinicians to tailor laser settings precisely, ensuring that energy is delivered efficiently to the intended tissue while sparing surrounding structures. By optimizing these parameters, practitioners can significantly reduce the risk of unintended thermal damage, thereby enhancing patient safety and minimizing potential side effects associated with laser therapy in oral surgery.

### 5.8. Comparisons with the Previous Literature and Unexpected Findings

In biological tissue, the predominant influence is due to the cellular and molecular structures that absorb and scatter light, ultimately limiting penetration depth [14].

Our findings showed an α of 144.8 cm^−1^ for 2940 nm and 142.3 cm^−1^ for 2780 nm, which closely align with Welch & van Gemert [26], who reported values exceeding 130 cm^−1^ for water-rich tissues. Similarly, 450 nm exhibited 26.8 cm^−1^, higher than the 20 cm^−1^ typically found in protein-rich chromophores [27].

Our findings are consistent with previous ex vivo research, including Kawamura et al.’s study [28], which demonstrated that Erbium lasers enable precise soft tissue ablation with minimal thermal damage compared to other systems such as electrosurgical tools. This trend was observed across most wavelengths in our study, except for KTP and Argon lasers, which exhibited different absorption behavior.

In addition to absorption, our measured thermal relaxation times (e.g., 0.2 s at 450–480 nm and 0.75 s at 810 nm) are in line with prior estimations for soft tissue cooling rates [15]. Compared to the findings reported by Lee et al. [27], our results demonstrate a slightly higher absorption coefficient (α) at 450 nm. This discrepancy may be attributed to variations in experimental conditions, such as differences in tissue hydration levels, sample thickness, tissue color, or preparation methods. These factors can significantly influence the optical properties of biological tissues, particularly in the blue light spectrum, and should be taken into account when interpreting and comparing absorption data across studies.

Overall, by evaluating 11 distinct wavelengths across the visible to mid-infrared spectrum, this study expands upon previous work and provides a broader comparative dataset for optimizing clinical laser selection.

Porcine soft tissue is abundant in protein, and the Argon laser is more readily absorbed by protein than the KTP laser [29]. This could elucidate why, in our study, the 480 nm laser exhibited greater absorption by porcine soft tissue compared to the 532 nm laser, contradicting the results typically documented in the literature based exclusively on hemoglobin absorption [29]. It is important to note that our study provides a global measurement of the optical behavior of oral tissue, rather than isolating the individual contributions of its chromophores such as hemoglobin, water, and collagen. This approach offers a more realistic representation of how lasers interact with composite biological tissues in vivo. Our findings highlight the limitations of relying solely on hemoglobin absorption data and underscore the importance of conducting whole-tissue studies when developing laser-based treatment protocols for oral applications.

### 5.9. Study Limitations

Due to ethical considerations, this study was conducted using ex vivo porcine gingival tissue, which, while structurally similar to human tissue, does not fully replicate the physiological complexity of living systems. The model lacks blood perfusion, active metabolism, and bodily fluids, all of which influence light absorption, thermal diffusion, and tissue response under clinical conditions. Additionally, differences in collagen content, hydration, and vascularization between porcine and human tissues may affect optical behavior and must be considered when extrapolating findings to humans.

Although our results suggest that certain wavelengths, such as 810 nm, may be effective for deep tissue penetration and photobiomodulation, clinical outcomes are highly dependent on tissue type, anatomical location, and therapeutic goals. For example, melanin- or water-rich tissues may exhibit different absorption characteristics, and thinner mucosa may require reduced fluence to prevent overtreatment. Furthermore, laser applications such as incision, coagulation, and PBM involve distinct energy thresholds and biological responses.

Therefore, these findings should be interpreted within the context of a controlled experimental setup, and further in vivo research is essential to validate the results across varied clinical scenarios and tissue types.

Despite this limitation, the results offer valuable insights into the interaction of light with pig oral tissue, which closely resembles human tissue [30]. To extrapolate the effects of laser to humans, further investigation through new pre-clinical and clinical trials is necessary [7] to confirm these findings and help refine laser use in dental and surgical settings.

Ultimately, each laser wavelength has its own strengths and limitations, depending on the specific clinical objective. A clear understanding of these differences enables clinicians to make evidence-based decisions when selecting the most appropriate wavelength for a given procedure, thereby optimizing treatment outcomes and ensuring patient safety.

## 6. Conclusions

This study confirms that laser wavelength significantly influences absorption, penetration depth, and thermal relaxation time in oral soft tissues. Erbium lasers demonstrated high absorption and shallow penetration. In contrast, diode lasers (810–980 nm) offered deeper penetration and a longer TRT. The 480 nm wavelength exhibited greater absorption in porcine gingival tissue compared to the 532 nm wavelength.

These findings support wavelength-based laser selection in both surgical and therapeutic dentistry. Future work should include in vivo validation, real-time dosimetry, and comparisons with other tissue types to refine clinical protocols.

## Figures and Tables

**Table 1:** Absorption coefficients (α, cm^−1^) of different wavelengths in porcine gingival tissue (*n* = 30). SD = standard deviation.

Wavelength (nm)	α (cm^−1^) and SD
450	26.8 ± 1.3
480	22.3 ± 1.1
532	18.9 ± 0.9
632	13.5 ± 0.7
810	9.6 ± 0.48
940	10.8 ± 0.5
980	10.4 ± 0.48
1064	10.0 ± 0.5
1341	14.0 ± 0.7
2780	142.3 ± 3.10
2940	144.8 ± 2.20

**Table 2:** The thermal relaxation time (TRT) of different wavelengths in porcine gingival oral tissues. SD = standard deviation.

Wavelength (nm)	TRT (s) and SD
450	0.25 ± 0.01
480	0.36 ± 0.021
532	0.50 ± 0.015
632	0.98 ± 0.05
810	1.93 ± 0.10
940	1.71 ± 0.091
980	1.85 ± 0.089
1064	1.76 ± 0.02
1341	0.90 ± 0.05
2780	0.008 ± 0.0004
2940	0.008 ± 0.00023

**Table 3:** Beam penetration depth (δ, mm) of different wavelengths in porcine gingival tissue. SD = standard deviation.

Wavelength (nm)	Beam Penetration Depth (mm)
450	0.37 ± 0.02
480	0.44 ± 0.018
532	0.52 ± 0.0195
632	0.74 ± 0.04
810	1.04 ± 0.044
940	0.93 ± 0.05
980	0.96 ± 0.046
1064	0.99 ± 0.052
1341	0.71 ± 0.044
2780	0.07 ± 0.0039
2940	0.06 ± 0.0028
